# Emotions of the pandemic: phenomenological perspectives

**DOI:** 10.1007/s11097-023-09926-x

**Published:** 2023-08-09

**Authors:** Luna Dolezal, Matthew Ratcliffe

**Affiliations:** 1 Wellcome Centre for Cultures and Environments of Health, University of Exeter, Queens Building, Queens Drive, Exeter EX4 4QH, UK; 2 Department of Philosophy, University of York, Heslington York YO10 5DD, UK

**Keywords:** COVID-19 pandemic, Emotional experience, Phenomenology, Social restrictions, Time, Online environments

## Abstract

This article provides an introduction to the special issue “Emotions of the Pandemic: Phenomenological Perspectives”. We begin by outlining how phenomenological research can illuminate various forms of emotional experience associated with the exceptional circumstances of the COVID-19 pandemic. In addition, we propose that a consideration of pandemic experience, in all its complexity and diversity, has the potential to yield wider-ranging phenomenological insights. We go on to discuss the thirteen contributions that follow, identifying common themes and points of complementarity.

The COVID-19 pandemic and the radical social restrictions that came with it affected our lives in a range of different ways. Many people reported experiences that were strange, disorientating, and difficult to comprehend and articulate. For example, there were accounts of pervasive but intangible feelings of loss, changes in the sense of time, and unfamiliar ways of encountering and interacting with others. Descriptions of altered experience encompassed a number of themes that phenomenologists have addressed at length—self, body, time, world, other people, mood, and emotion. Hence, the prospect arises of casting light on the nature of pandemic experiences by drawing on insights from phenomenology, while at the same time gaining novel insights into the structure of human experience by reflecting on the full range of pandemic experiences.

In this special issue, we embark upon such an enquiry, focusing specifically on the theme of *emotional* experience (broadly construed). Given that the pandemic and associated public health measures, such as lockdowns and social distancing, pro-foundly the fabric of social and personal life, they were associated with a range of emotions. Experiences reported as prevalent during the pandemic included the likes of fear, anxiety, bewilderment, disorientation, outrage, boredom, anger, shame, grief, and loss. Although the emotional landscape seemed at times to be predominantly negative, there were also those who reported positive emotional experiences, including feelings of relief, hope, gratitude, contentment, and solidarity, as well as reductions in anxiety and stress.

Hence, there is no singular account to be had of “pandemic emotional experience”. The pandemic affected individuals in different and sometimes contrasting ways, for a multitude of reasons. In the early stages of the pandemic, some of us were able to work from home, while others continued to work on the “frontline”, at considerable risk to their health. For some, radical social restrictions, such as national lockdowns, provided opportunities to reflect, slow down, and spend time with family. Others, however, faced highly distressing circumstances, such as illness, grief, financial hardship, social isolation, loneliness, and anxiety. One important factor here was relative social privilege. Those living with marginalization, racism, poverty, ill health, disability and other social inequalities were more likely to be negatively affected by the pandemic—socially, emotionally and physically.

Even so, what at least seems plausible is that the majority of pandemic experiences involved *some form* of significant phenomenological disruption or change. The taken-for-granted order of social, personal, and professional life was rendered contingent, uncanny, or unstable, in ways that have the potential to yield phenom-enological insights. Indeed, it has been suggested that we can *do* phenomenology by reflecting on various different kinds of disturbances in the structure of experience, which disrupt and in so doing make explicit aspects of experience that are more usually presupposed (Ratcliffe, 2015). As Merleau-Ponty famously put it, distinctively phenomenological forms of reflection involve a loosening of the “intentional threads that connect us to the world”, whereby we glimpse underlying phenomenological achievements that are more usually taken for granted (Merleau-Ponty, 1945/2012, lxxvii).

Hence, there is the possibility that investigating pandemic experience will not only prove illuminating with respect to familiar categories of emotion. In addition, as the contributions to this special issue demonstrate, the exceptional circumstances faced by many people were associated with forms of affective experience that are unusual and—in some instances— even lacking in established names. They thus bring to light aspects of our emotional lives that might otherwise have remained unavailable to explicit philosophical reflection.

In order to investigate the prospects for mutually illuminating interaction between phenomenological research and reflection upon circumstances and associated experiences that arose during the pandemic, this special issue brings together researchers involved in four major collaborative research projects:

**The Pandemic Experience Survey** (Froese; James; Koshkina; Ratcliffe)
**Shame and Medicine Project** (Dolezal; Rose)
**Grief: A Study of Human Emotional Experience** (Hughes; Millar; Ratcliffe; Richardson)
**Antagonistic Political Emotions** (Osler; Tietjen)^1^

We also invited additional contributors with complementary areas of expertise.

The articles featured here address a range of emotional and wider affective experiences, while also exploring various contexts that were uniquely afforded by the pandemic and the exceptional circumstances that many people lived through. It is important to concede from the outset that we cannot do full justice to the complexity and diversity of people’s emotional lives during this time. The articles in this issue are by no means exhaustive of the emotions and circumstances that resulted from the pandemic. Nevertheless, we have at least sought to identify some key themes, while also illustrating something of the potential for dialogue between phenomenological research and ongoing efforts to comprehend what has taken place.

In the first article, “Becoming Anonymous: How Strict COVID-19 Isolation Protocols Impacted ICU Patients”, **Allan Køster**, draws on a combination of interviews, observations, and phenomenological reflections in order to articulate the profound and prolonged departure from everyday social life undergone by COVID-19 patients in Intensive Care Units. He suggests that those placed in such situations undergo substantial changes in the overall structure of experience, involving a form of affective experience that is utterly unfamiliar to many people, lacks an established name, and is consequently very difficult to describe. Køster characterizes it in terms of “becoming anonymous”. One loses the sense of being a distinctive subject of experience or person, as the pre-personal, material body becomes conspicuous in unfamiliar ways. Køster suggests that such extraordinary experiences make salient how the sense of being a particular person is ordinarily scaffolded by interpersonal and social relations that we take for granted.

The next article, “‘We’re protecting them to death’—A Heideggerian Interpretation of Loneliness among Older Adults in Long-Term Care Facilities during COVID-19”, by **Kevin Aho**, is similarly concerned with the theme of social privation and its effects upon emotional experience. Aho addresses the experiences of care home residents who were deprived of social interactions for lengthy periods. Drawing on Heidegger’s analysis of mood (*Stimmung*), he emphasizes the profundity of the loneliness that many people experienced under these conditions. Deprivation of familiar routines and regular interpersonal contact can, Aho suggests, amount to an all-pervasive sense of disconnection and to an experiential world bereft of practical significance. Thus, in interpreting talk of “loneliness” during the pandemic, we can draw on phenomenological resources in order to better understand what certain experiences involve—a privation of self and world, as opposed to a more superficial form of affective experience that arises against the backdrop of an intact world.

In the third article, “Phenomenological Reflections on Grief during the COVID-19 Pandemic”, **Matthew Ratcliffe** further investigates the relationships between social isolation and emotional experience, by considering some of the ways in which social restrictions affected people’s experiences of bereavement. First of all, he suggests that grief is not an episodic emotion or a longer-term mood, but instead a temporally extended emotional process. This process, he adds, is shaped and scaffolded by interpersonal interactions, situated within the context of a larger social world. Thus, deprivation of familiar social routines and interpersonal relations can affect grief in a number of ways. In some cases, it led to a form of grief that was lacking in movement and involved an enduring sense of unreality.

Ratcliffe further suggests that experiences of bereavement-grief during the pandemic were inextricable from wider experiences of loss, spanning all that was no longer possible. This theme is taken up by **Louise Richardson and Becky Millar** in their article “Grief and the Non-death Losses of COVID-19”. They ask whether or not such experiences should be taken to involve “grief”, in a literal sense of the term, or whether they are importantly different from the kind of grief we experience in response to the death of a loved one. They propose that emotional experiences of death and non-death losses resemble one another in several important ways, thus supporting a broad conception of grief. Moreover, they argue that grief over seemingly trivial losses should sometimes be taken seriously. Grief involves experiencing the loss of pervasive networks of possibilities, which were central to who one was and what one strived to become. Such losses can be expressed in terms of numerous other losses that are more specific and concrete in nature. Hence, seemingly trivial losses can be indicative of larger, less tangible losses that are of considerable significance to a person.

**Emily Hughes’s** article “Meaninglessness and Monotony in Pandemic Boredom” also addresses the theme of the profundity of emotional changes during the pandemic, by turning to the widely reported phenomenon of pandemic boredom. Drawing on Heidegger’s influential account of boredom, Hughes explores whether pandemic boredom involves only a sense of contingent meaningless, situated within the context of the everyday world, or instead an existential confrontation with absolute meaninglessness, thus unpacking distinctions drawn by Heidegger. In order to illuminate the experiences of temporal disruption, emptiness, restlessness, frustration, weariness, and indifference that were frequently associated with boredom during the pandemic, Hughes draws on testimonies collected via the Pandemic Experience Survey (Froese et al., 2021). In so doing, she highlights the complexities of trying to generalise about pandemic experiences. Boredom was experienced in a range of ways and had varying significance for people; it was profoundly “situative”. Yet pandemic boredom was also inflected with existential significance, thus problematising the distinction between situational and existential boredom in Heidegger’s work.

Profound boredom, anxiety, and grief are not just experienced *in* time; they also involve distinctive ways of experiencing time itself. The theme of pandemic time is explored further by **Pablo Fernandez Velasco**, **Bastien Perroy**, **Umer Gurchani**, and **Roberto Casati**, in their article, “Lost in Pandemic Time: A Phenomenological Analysis of Temporal Disorientation During the COVID-19 Crisis”. They focus on feelings of temporal *disorientation* during the pandemic and proceed to identify a number of ways in which people’s experiences of time were altered due to pandemic restrictions and consequent disruption of familiar activities. For many of us, there was a pronounced contrast between the times of “before” and “after”. Time was also experienced as passing fast, slowly, and sometimes both. With the monotony of lockdowns, time seemed curiously impoverished, undifferentiated. Some experienced a growing inability to see beyond this—to imagine and contemplate alternative possibilities. In light of all this, Fernandez Velasco et al. propose that, without social scaffolding, temporal experience loses structure; we become temporally disoriented. They identify the overarching theme as that of “suspended time”. It was as though time itself had been stopped, parenthesized, or put on hold.

In their article, “On Being Stuck: The Pandemic Crisis as Affective Stasis”, **Fabian Bernhardt** and **Jan Slaby** similarly focus on an experience of time as lacking in movement. With the term “affective stasis”, they identify a form of emotional experience that lacks an established name in modern English. There are, they propose, two inextricable aspects to this—a feeling of being stuck and the inchoate anticipation of something threatening, as though this were the “calm before the storm”. To characterize affective stasis, they turn to the connotations of “stasis” in Ancient Greek, a term that encompasses both lack of motion and radical disruption. Their account also relates the phenomenology of emotion to political considerations. For Bernhardt and Slaby, affective stasis is not just a matter of how an individual feels; it is also inextricable from the socio-political context within which emotional experiences arise. Their account thus illustrates how phenomenological engagement with pandemic experience can be a way of revealing emotional phenomena that are otherwise elusive. Furthermore, these phenomena draw attention to the challenge of integrating phenomenological and scientific work on emotion at the level of the individual with an indispensable political perspective on those same phenomena.

Another conspicuous theme throughout the pandemic—often mentioned in reports of boredom, disorientation, time standing still, and the like—was that of moving “online”. People were not simply deprived of certain social opportunities and activities. Often, they were presented with more or less effective online alternatives. An important and closely related topic to consider is that of emotion regulation. As noted by Ratcliffe, grief can be dysregulated by a lack of social and interpersonal interaction. Similar points apply to emotional experiences more generally. In their contribution, “From Tech to Tact: Emotion Dysregulation in Online Communication during the COVID-19 Pandemic”, **Mark James, Natalia Koshkina, and Tom Froese** draw on the findings of the Pandemic Experience Survey (as described by Froese et al., 2021) in order to develop a nuanced account of how online social environments can disrupt the regulation of emotional experience. In so doing, they distinguish a number of interrelated ways in which online social environments render emotion regulation “precarious”. They add that the likelihood of emotion dysregulation depends to a considerable degree on the adequacy of technological resources and the competences of those using them. One important consideration is what they refer to as “digital tact”, something that we come to develop ourselves and recognize in others as we establish and adapt to norms for online interaction. James et al. further emphasize the practical importance of these issues, given the likelihood of a lasting shift towards online interactions in the wake of the pandemic.

As the development of “digital tact” indicates, it is important to acknowledge that online resources not only render emotion regulation precarious; they can also provide novel opportunities for regulation. For instance, “moving online” and conducting social and professional life in virtual spaces may have mitigated certain forms of unpleasant emotional experience involving bodily conspicuousness. In her article “Healing Online? Social Anxiety and Emotion Regulation in Pandemic Experience”, **Anna Bortolan** discusses social anxiety disorder and how the experience of Internet-based communications afforded an alleviation of anxiety for some. Bortolan emphasizes how for those living with social anxiety, social interactions can disrupt bodily experience as a result of heightened bodily self-consciousness resulting from a fear of negative judgement from others. She highlights how the modifications to inter-subjective experience made possible by Internet-mediated interactions can weaken the symptoms of social anxiety, by affording individuals more control over their self-presentation and social experience. Although the move to online spaces was often characterized as diminishing the quality of social interaction, causing negative experiences such as alienation, isolation, and “Zoom-fatigue”, Bortolan thus emphasizes the need for a more balanced treatment, one that also acknowledges positive outcomes of the increased use of online spaces and virtual communication.

**Lucy Osler** is similarly concerned with how online environments can regulate, dysregulate, and even transform emotional life. In her article, “WTF?! COVID-19, Indignation, and the Internet”, Osler focuses specifically on the experience of indignation, an under-theorized form of anger that involves reacting to moral offence with incredulity. In addition to addressing what indignation consists of, she develops a detailed account of what indignation *does*, exploring the relationships between indignation, moral disapproval, virtue-signaling, and the fostering of social connection. According to Osler, certain features of online environments and, more specifically, social media platforms encourage the proliferation of indignation. In addition, they can promote its employment to express one’s own moral certainty and superiority, in ways that are sometimes insensitive to the complexities of social situations. On the other hand, indignation can also play important roles in making salient and challenging genuine injustices. So, indignation is not simply felt; it is both “performed” and “received” in ways that are shaped and regulated by features of social environments.

The interpersonal dynamics of emotional disapproval are also considered by **Luna Dolezal and Arthur Rose**, whose article “A Sartrean Analysis of Pandemic Shaming” explores how COVID-19 came to be characterized as the “public-shaming pandemic”. Shaming circulated liberally in both online and offline spaces, in ways that served informally to police public health rules and encourage “proper” pandemic behaviour. Dolezal and Rose’s analysis turns to Jean-Paul Sartre’s accounts of “the look” and shame, using his claims regarding the antagonistic nature of interpersonal relations as illustrative of the suspicious and untrustworthy atmosphere that characterized some social and community spaces, both online and offline. With particular focus on Sartre’s well-known voyeur example, they illustrate the iterative structure of pandemic shaming acts—how pandemic shaming was often accompanied by shame backlashes, where the shamer was shamed by onlookers who did not agree with their epistemic or moral authority. Dolezal and Rose conclude with some socio-historical reflections on how the German occupation of Paris during World War II may have influenced Sartre’s philosophical account of self-other relations. By turning to Sartre’s essay “Paris Under Occupation”, and drawing parallels with the war metaphorsaturated COVID-19 landscape, Dolezal and Rose argue that experiences of shame and the impulse towards acts of shaming are more likely in conditions of uncertainty and vulnerability.

Also considering negative interpersonal emotions, **Shiloh Whitney’s** article “Anger and Uptake” explores how anger at racial injustice became transformative during the pandemic. Whitney discusses how, following the murder of George Floyd in May 2020, there was a “breakthrough narrative”, whereby anger directed at racial injustice became apt and celebrated during the pandemic, rather than dismissed as problematic or even criminal. Anger, Whitney argues, became a mainstream force in social politics, where the righteous rage of protestors in the Black Lives Matter movement was instrumental in shifting the social and moral orientation of others. In other words, this anger was given “uptake”, as Whitney argues with reference to Marilyn Frye’s work. Drawing on Merleau-Ponty’s phenomenology of the body schema, Whitney describes the second-person experience of affective intentionality, which can involve being reoriented by the experience of another’s anger. But uptake can also fail, as when pre-existing interpretative frameworks mean that anger is dismissed in our shared situation and affective milieu. Whitney’s account highlights the centrality of intercorporeality in shared emotional experiences, where emotions are not merely cognitive in nature but embodied and expressive, always present in the spaces and situations between self and other.

The final contribution in this special issue, “Feeling and Performing ‘the Crisis’: On the Affective Phenomenology and Politics of the Corona Crisis” by **Ruth Rebecca Tietjen**, steps back from specific forms of emotional experience, in order to provide a wider-ranging consideration of what it is to experience a *crisis* (something that spans various aspects of experience addressed by other contributors). In addition to our experiencing more specific emotions during the pandemic, Tietjen suggests that it *felt like* something to be in a crisis. To capture this, we need to consider the range of affective experiences had by individuals and how those experiences relate to one another. However, in order to appreciate what it is to find ourselves in the midst of a collective crisis, we must also acknowledge the *political* dimensions of crises and the manner in which they contribute to affective experience. As certain crisis-experiences are inextricably affective and political in nature, Tietjen observes that they can feed into critique and at the same time be subjected to critique themselves.

Taken together, these articles yield important insights into emotional experience during the pandemic. They demonstrate a range of ways in which phenomenological and interdisciplinary research is profoundly relevant not only when seeking to understand lived experience in general, but also in highlighting important experiential considerations in the realms of healthcare, technology and politics. This special issue points not only to the challenge of integrating phenomenology and the cognitive sciences, but also to that of reconciling both with the political dimensions of emotional experience.

## Declarations

1 Websites and funders details for the four projects are as follows: Pandemic Experience Survey (JSPS Topic Setting Program to Advance Cutting Edge Humanities and Social Sciences Research; Grant Ref. JPJS00120350377): https://www.frontiersin.org/articles/10.3389/fpubh.2021.725506/full; Shame and Medicine Project (Wellcome Trust; Grant Ref. 217,879/Z/19/Z): https://shameandmedicine.org/. Under the umbrella of the Shame and Medicine Project, Dolezal and Rose also worked on the Scenes of Shame and Stigma in COVID-19 Project (AHRC; Grant Ref. AH/V013483/1): https://shameandmedicine.org/covid-19/; Grief: A Study of Human Emotional Experience (AHRC; Grant Ref. AH/T000066/1):https://www.griefyork.com; Antagonistic Political Emotions: An Integrative Philosophical Analysis (Austrian Science Fund; Grant Ref. P-32,392-G): https://antagonisticpoliticalemotions.squarespace.com/our-people.
